# First mitochondrial genome of the Amazonian marsh rat *Holochilus sciureus* Wagner 1842 (Rodentia, Cricetidae, Sigmodontinae)

**DOI:** 10.1080/23802359.2022.2107956

**Published:** 2022-08-29

**Authors:** Susette Castañeda-Rico, Jesús E. Maldonado, Robert C. Dowler, Cody W. Edwards

**Affiliations:** a Smithsonian-Mason School of Conservation, Front Royal, VA, United States; b Center for Conservation Genomics, Smithsonian National Zoo and Conservation Biology Institute, Washington, DC, United States; c Department of Biology, George Mason University, Fairfax, VA, United States; d Department of Biology, Angelo State University, San Angelo, TX, United States

**Keywords:** *Holochilus*, mitogenome, museum specimens, Sigmodontinae, South America

## Abstract

The Amazonian marsh rat, *Holochilus sciureus*, is a member of the subfamily Sigmodontinae, the second-largest subfamily of muroid rodents, with 410 species and ca. 84 genera in 12 tribes. This semiaquatic rodent is distributed in South America and is of great economic and epidemiological importance. In this study, we obtained the first mitochondrial genome of the genus *Holochilus* obtained from a tissue sample associated with a museum voucher specimen. The generated mitogenome sequence of *H. sciureus* is 16,358 bp length. It comprises a control region and a conserved set of 37 genes encoding for 2 rRNA genes, 22 tRNA genes and 13 protein-coding genes. We conducted a phylogenetic analysis that included *H. sciureus* and the only five other published mitochondrial genomes of this poorly studied subfamily of rodents.

## Introduction

The genus *Holochilus*, a sigmodontine genus of large, herbivorous, and semiaquatic rodents, is broadly distributed and inhabits several environments throughout South America (Musser and Carleton 2005). *Holochilus sciureus* is distributed in the lowlands of the Guianas, Peru, Bolivia, and Brazil (D’Elía et al. 2015; Prado et al. 2021b). It is considered a member of a species complex, whose taxonomy and phylogenetic relationships have undergone several changes including the composition and names of species (Prado et al. 2021a; 2021b). *H. sciureus* has an economic and epidemiological importance since it is considered a pest in rice and sugar cane fields (Eiris and Barreto 2009) and as a sentinel species as it is a host for parasites causing severe human diseases (da Silva-Souza et al. 2019).

In this study, we sequenced the first publicly available mitogenome of the genus *Holochilus* from a tissue sample, on loan, of *H. sciureus* associated with a museum voucher specimen, both deposited at the Museum of Vertebrate Zoology, University of California, Berkeley with catalog number MVZ:Mamm:190356 (https://mvz.berkeley.edu/mvzmamm/ Chris Conroy, ondatra@berkeley.edu). The specimen was collected in 1991 in Brazil, Amazonas, Eirunepé river Juruá (-6° 37′ 59.88″ S, −69° 52′ 0.012″ W).

We extracted DNA from a tissue sample using the DNeasy^®^ Blood and Tissue Kit (QIAGEN, Inc., Valencia, CA, US). We quantified the DNA extraction using a Qubit^®^ (Thermo Fisher) fluorometer with a 1x dsDNA HS assay kit. We used the single tube library preparation method described by Carøe et al. (2018) and dual indexing PCR with TruSeq-style indices (Meyer and Kircher 2010) using Kapa HiFi HotStart Ready Mix (Roche Sequencing). We used the myBaits^®^ UCE Tetrapods 5Kv1 kit (Arbor Biosciences) to perform target enrichment. We amplified the post-enrichment UCE library with 14 cycles of PCR using universal Illumina primers and Kapa HiFi Hotstart Ready Mix, and sequenced on a NovaSeq 6000 (Illumina, Inc., San Diego, CA, US) at the Oklahoma Medical Research Foundation, Oklahoma City (combined with samples from other projects).

We removed low-quality reads and adapter contamination using Trim Galore 0.6.5 (https://github.com/FelixKrueger/TrimGalore) and Prinseq-lite v0.20.4 (Schmieder and Edwards, 2011) to remove exact duplicates (-derep1,4). We mapped the cleaned reads to a reference (*Melanomys caliginosus* GenBank MH939287) using Geneious algorithm in Geneious Prime^®^ 2021.2.2 with default parameters, and aligned using MAFFT 7.45 plug-in (Katoh and Standley 2013). To rule out the presence of nuclear copies of mitochondrial genes (NUMTs), we transferred annotations from the reference genome and translated all protein-coding genes (PCGs) to check for frame shifts or stop codons.

The generated complete mitogenome sequence of *H. sciureus* is 16,358 bp in length (GenBank accession number OL685394), which covers 100% of the reference genome sequence. The average sequencing depth was 1,700x with 238,757 reads mapped to the reference genome. The sequence contains the standard features present in a vertebrate mitochondrial genome including one non-coding *control region*, 2 rRNA genes (*12S rRNA* and *16S rRNA*), 22 tRNA genes, and 13 PCGs including ones for NADH dehydrogenase (*ND1, ND2, ND3, ND4, ND4L, ND5* and *ND6*), ones from cytochrome c oxidase (*COX1, COX2,* and *COX3*), ATP synthase (ATP6 and *ATP8*) and *cytochrome b* gene. The base composition was 34.2% A, 25.2% C, 12.3% G, 28.4% T and the GC content was 37.4%.

**Figure 1.**
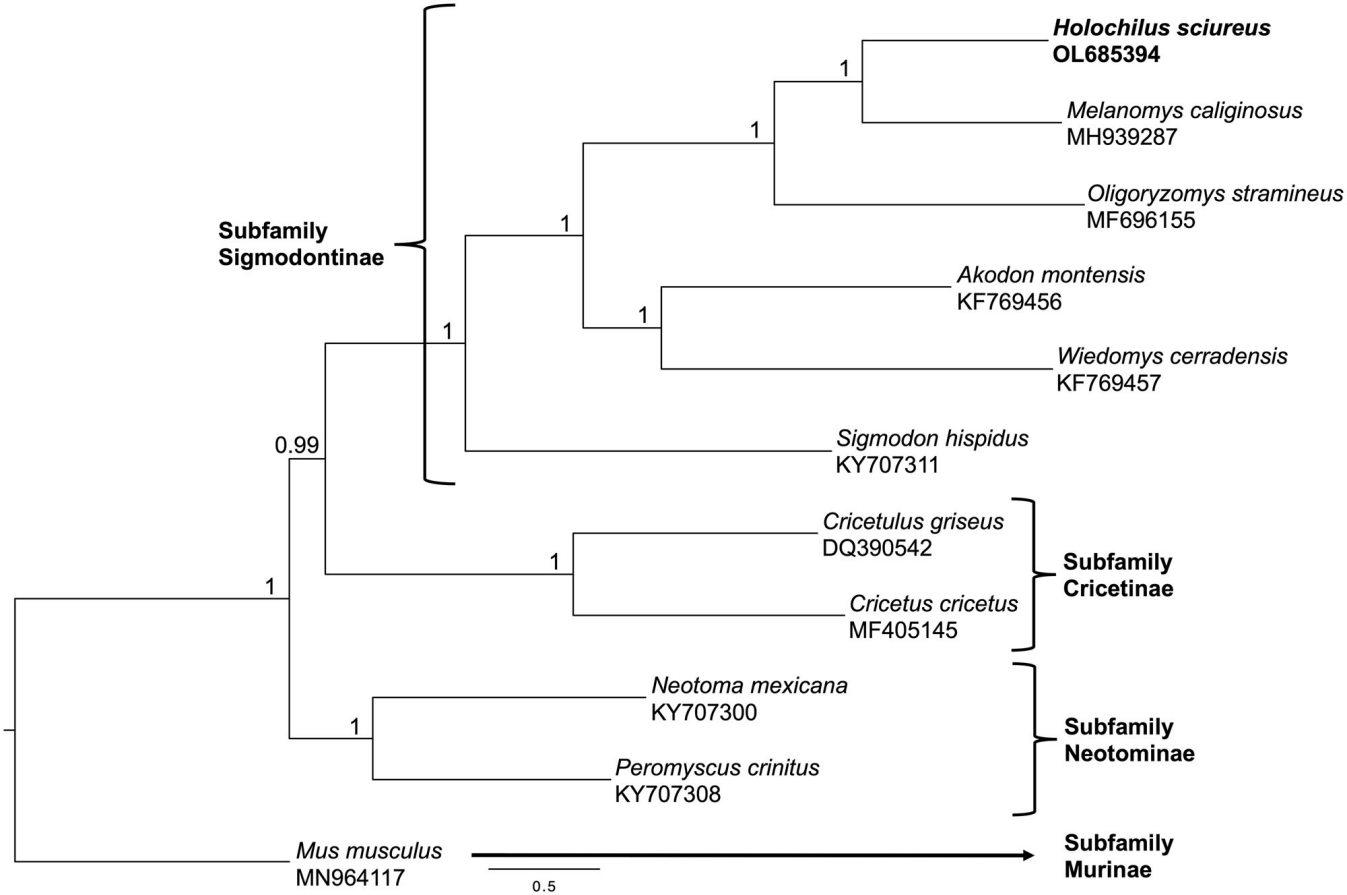
Bayesian Inference phylogeny of Sigmodontinae rodents using complete mitochondrial genomes. Nodal support is provided with posterior probabilities values.

A Bayesian Inference (BI) analysis, including all the Sigmodontinae mitogenomes available in GenBank (accessed on 15 November 2021) and five outgroups, was performed using MrBayes 3.2.6 (Ronquist et al. 2012) under the best model and partition scheme (by gene and codon position) selected by PartitionFinder 2.1.1 (Lanfear et al. 2016). The analysis ran for 50 million generations sampling every 1,000 generations with a burn-in of 25%. Output parameters were visualized using Tracer v1.7.1 (Rambaut et al. 2018) to check for convergence between runs. The resulting phylogenetic tree was visualized using FigTree v1.4.4 (http://tree.bio.ed.ac.uk/software/figtree/). Our phylogenetic hypothesis based on a BI analysis supports the placement *H. sciureus* as sister to *M. caliginosu*s and within a clade that includes the few other members of the Sigmodontinae sequenced to date (Figure 1).

Species delimitation has been challenging for the *H. sciureus* complex. Two recent studies conducted by Prado et al. (2021a; 2021b) using morphological and morphometric characters, and nuclear genomic data (Single Nucleotide Polymorphisms -SNPs) redefined the species boundaries within *H. sciureus* sensu Gonçalves et al. (2015) into three different species (*H. sciureus, H. nanus* and *H. oxe spp. nov*.). However, phylogenetic analysis using complete mitochondrial genomes have shown to be invaluable for understanding the evolutionary history and taxonomy of several taxa (Duchêne et al. 2011; Abramson et al. 2021). Hence, we expect that the mitochondrial genome generated in this study will contribute to future genomic studies that aim to resolve the taxonomy and phylogenetic relationships within the genus *Holochilus* as well as adding to the few mitochondrial genomes available for the subfamily Sigmodontinae.

## Data Availability

The mitochondrial genome sequence is available in GenBank at https://www.ncbi.nlm.nih.gov/genbank/ under the accession number OL685394. The associated BioProject, BioSample and SRA numbers are PRJNA818347, SAMN26856163, and SRR18404174, respectively.
